# Supplementary material for: Effectiveness and Feasibility of Workplace-Based Mental Health Interventions for University Academic Staff: A Systematic Review
Source: Int J Environ Res Public Health. 2025 Nov 26;22(12):1787. doi: 10.3390/ijerph22121787 (PMC12732473; doi:10.3390/ijerph22121787)
Supplement: Supplementary file 1 [file ijerph-22-01787-s001.zip › Supplementary File 2 - Search Strategy.pdf]

**Supplementary File 2:**

*Searches on all databases were limited to **English language** and items published between **01 January 2023 and 12 December 2023.***

**<u>Search strategies for databases:</u>**

**EBSCO (Used to search ERIC, Medline and PsycInfo)**

1. **TI ( ((universit* OR college* OR "higher education" OR postsecondary OR tertiary) N5 (staff OR faculty OR academic* OR employee* OR worker* OR administrator* OR lecturer* OR professor* OR adjunct* OR educator* OR "faculty member*" OR instructor* OR preceptor* OR researcher*)) ) OR AB ( ((universit* OR college* OR "higher education" OR postsecondary OR tertiary) N5 (staff OR faculty OR academic* OR employee* OR worker* OR administrator* OR lecturer* OR professor* OR adjunct* OR educator* OR "faculty member*" OR instructor* OR preceptor* OR researcher*)) )**
2. **TI ( ((promot* OR enhanc* OR multicomponent OR "multi-component" OR multimodal OR multi-modal OR "behaviour change*" OR "behavior change*") N10 (program OR programme OR programs OR programmes OR programming OR intervention* OR course* OR initiative* OR framework* OR strategy OR strategies OR training OR internet OR digital OR online OR workplace* OR institution* OR organisation* OR organization* OR occupation*)) ) OR AB ( ((promot* OR enhanc* OR multicomponent OR "multi-component" OR multimodal OR multi-modal OR "behaviour change*" OR "behavior change*") N10 (program OR programme OR programs OR programmes OR programming OR intervention* OR course* OR initiative* OR framework* OR strategy OR strategies OR training OR internet OR digital OR online OR workplace* OR institution* OR organisation* OR organization* OR occupation*)) )**
3. **TI ( (((mental OR psychological OR emotional OR psychosocial) N (health OR wellbeing OR "health and wellbeing")) OR wellness OR empowerment OR flourishing OR resilience OR "self-compassion" OR "self-esteem" OR "self-efficacy" OR depression OR anxiety OR distress OR stress OR loneliness OR insomnia OR "sleep deprivation" OR "social dysfunction" OR burnout) ) OR AB ( (((mental OR psychological OR emotional OR psychosocial) N (health OR wellbeing OR "health and wellbeing")) OR wellness OR empowerment OR flourishing OR resilience OR "self-compassion" OR "self-esteem" OR "self-efficacy" OR depression OR anxiety OR distress OR stress OR loneliness OR insomnia OR "sleep deprivation" OR "social dysfunction" OR burnout) )**
4. **S1 AND S2 AND S3**
5. **S1 AND S2 AND S3 (LIMITERS Publication date 2003-01-01 to 2023 -12 - 31**
6. **Narrow by language : English**

**Web of Science**

1. **TI=(((universit* OR college* OR "higher education" OR postsecondary OR tertiary) NEAR/5 (staff OR faculty OR academic* OR employee* OR worker* OR administrator* OR lecturer* OR professor* OR adjunct* OR educator* OR "faculty member*" OR instructor* OR preceptor* OR researcher*)))**
2. **AB=(((universit* OR college* OR "higher education" OR postsecondary OR tertiary) NEAR/5 (staff OR faculty OR academic* OR employee* OR worker* OR administrator* OR lecturer* OR professor* OR adjunct* OR educator* OR "faculty member*" OR instructor* OR preceptor* OR researcher*)))**
3. **(TI=(((promot* OR enhanc* OR multicomponent OR "multi-component" OR multimodal OR multi-modal OR "behaviour change*" OR "behavior change*") NEAR/10 (program OR programme OR programs OR programmes OR programming OR intervention* OR course* OR initiative* OR framework* OR strategy OR strategies OR training OR internet OR digital OR online OR workplace* OR institution* OR organisation* OR organization* OR occupation*)))) OR AB=(((promot* OR enhanc* OR multicomponent OR "multi-component" OR multimodal OR multi-modal OR "behaviour change*" OR "behavior change*") NEAR/10 (program OR programme OR programs OR programmes OR programming OR intervention* OR course* OR initiative* OR framework* OR strategy OR strategies OR training OR internet OR digital OR online OR workplace* OR institution* OR organisation* OR organization* OR occupation*)))**
4. **(TI=((((mental OR psychological OR emotional OR psychosocial) NEAR (health OR wellbeing OR "health and wellbeing")) OR wellness OR empowerment OR flourishing OR resilience OR "self-compassion" OR "self-esteem" OR "self-efficacy" OR depression OR anxiety OR distress OR stress OR loneliness OR insomnia OR "sleep deprivation" OR "social dysfunction" OR burnout))) OR AB=((((mental OR psychological OR emotional OR psychosocial) NEAR (health OR wellbeing OR "health and wellbeing")) OR wellness OR empowerment OR flourishing OR resilience OR "self-compassion" OR "self-esteem" OR "self-efficacy" OR depression OR anxiety OR distress OR stress OR loneliness OR insomnia OR "sleep deprivation" OR "social dysfunction" OR burnout))**
5. **#1 OR #2**
6. **#3 AND #4 AND #5**
7. **#3 AND #4 AND #5** and **2003** or **2005** or **2004** or **2006** or **2007** or **2008** or **2009** or **2010** or **2011** or **2012** or **2013** or **2014** or **2015** or **2016** or **2017** or **2018** or **2019** or **2020** or **2021** or **2022** or **2023** (Publication Years)

SCOPUS:

( TITLE-ABS-KEY ( universit* OR college* OR "higher education" OR postsecondary OR tertiary AND staff OR faculty OR academic* OR employee* OR worker* OR administrator* OR lecturer* OR professor* OR adjunct* OR educator* OR "faculty member*" OR instructor* OR preceptor* OR researcher* ) ) AND ( TITLE-ABS-KEY ( promot* OR enhanc* OR multicomponent OR "multi-component" OR multimodal OR multi-modal OR "behaviour change*" OR "behavior change*" program OR programme OR programs OR programmes OR programming OR intervention* OR course* OR initiative* OR framework* OR strategy OR strategies OR training OR internet OR digital OR online OR workplace* OR institution* OR organisation* OR organization* OR occupation* ) ) AND ( TITLE-ABS-KEY ( mental OR psychological OR emotional OR psychosocial AND health OR wellbeing OR "health and wellbeing" OR wellness OR empowerment OR flourishing OR resilience OR "self-compassion" OR "self-esteem" OR "self-efficacy" OR depression OR anxiety OR distress OR stress OR loneliness OR insomnia OR "sleep deprivation" OR "social dysfunction" OR burnout ) ) AND ( TITLE ( staff OR employe* OR personnel OR "work force" OR workplace OR worksite OR worker* ) ) AND ( TITLE ( ( universit* OR academic ) ) )
